# Factors contributing to ice nucleation and sequential freezing of leaves in wheat

**DOI:** 10.1007/s00425-021-03637-w

**Published:** 2021-05-20

**Authors:** D. P. Livingston, A. Bertrand, M. Wisniewski, R. Tisdale, T. Tuong, L. V. Gusta, T. Artlip

**Affiliations:** 1 USDA-ARS and North Carolina State University, Raleigh, NC 27607 USA; 2 Quebec Research and Development Centre, Agriculture and Agri-Food Canada, 2560 Hochelaga Boulevard, Québec, QC G1V 2J3 Canada; 3 USDA-ARS, Appalachian Fruit Research Station, Kearneysville, WV USA; 4 Department of Plant Science, Univ Saskatchewan, Saskatoon, Canada; 5 USDA-ARS, Appalachian Fruit Research Station, Kearneysville, WV 25430 USA; 6 Present Address: Virginia Polytechnic Institute, Blacksburg, VA 24061 USA

**Keywords:** Amino acids, Carbohydrates, Freeze initiation, Fructan, Infrared thermography, Leaf anatomy, Microflora, Sucrose, Supercooling

## Abstract

**Main conclusion:**

Anatomical, metabolic and microbial factors were identified that contribute to sequential freezing in wheat leaves and likely contribute to supercooling in the youngest leaves and potentially meristematic regions.

**Abstract:**

Infrared thermography (IR) has been used to observe wheat leaves freezing independently and in an age-related sequence with older leaves freezing first. To determine mechanisms that might explain this sequence of freezing several analytical approaches were used: (1) The size of xylem vessels, in proximity to where freezing initiated, was measured to see if capillary freezing point depression explained sequential freezing. The sequence of freezing in the four youngest leaves was correlated, with the largest vessels freezing first. (2) Carbohydrate and amino acids were analyzed to determine if solute concentrations as well as interactions with membranes explained the freezing sequence. Sucrose was highly correlated to the freezing sequence for all leaves suggesting a prominent role for this sugar as compared to other simple sugars and fructans. Among individual free amino acids proline and serine were correlated to the freezing sequence, with younger leaves having the highest concentrations. (3) Microflora within and on leaf surfaces were determined to measure potential freezing initiation. Levels of bacteria and fungi were correlated to the freezing sequence for all leaves, and species or genera associated with high ice nucleation activity were absent in younger leaves. Moisture content and transcript expression of ice binding proteins were also measured. As expected, our results show that no single mechanism explains the freezing sequence observed via infrared analyses. While these multiple mechanisms are operative at different levels according to the leaf age, they seem to converge when it comes to the protection of vital meristematic tissues. This provides potential phenotypic characters that could be used by breeders to develop more winter-hardy genotypes.

## Introduction

Winter cereals such as wheat, barley, rye and oats are planted in the fall and harvested the following spring or summer (or fall in northern regions) depending on growing conditions. In mid-Atlantic states in the US this avoids hot and dry conditions in the summer frequently encountered by spring planted crops. Fall planting can result in a 20–40% higher yield than that of a spring-planted crop (Shands and Chapman 1961). In addition, depending on the growing region, winter crops are harvested several weeks earlier than their spring-planted counterparts, which gives growers the option to plant a second crop.

The major detriment to growing a fall-seeded crop is its susceptibility to freezing conditions during winter. Much of the complexity involved in freezing in plants has been underscored by observations of freezing using infrared (IR) thermographic imaging that can monitor ice propagation between and within plants (Wisniewski et al. 1997, 2015; Livingston 2018). This technology has demonstrated the role of different factors in extrinsic and intrinsic ice nucleation, including the role of ice-nucleating bacteria, thick, waxy cuticles, stomata location, and antifreeze proteins.

IR thermography has revealed that freezing in grasses, in both controlled (Livingston et al. 2016) and natural conditions (Livingston et al. 2018), begins at the base of the plant and propagates upwards. This was a counter-intuitive observation considering that the top of the plant is coldest when freezing is initiated (Single 1964; Livingston et al. 2018). IR thermography also demonstrated that freezing begins in the vascular system of stems and proceeds into leaves (Hacker and Neuner 2007). This is contrary to the general assumption that ice propagates into leaves through wounds in the cuticle or through stomata or hydathodes (Olien and Smith 1981; Pearce and Fuller 2001; Griffith et al. 2005).

In addition to plants freezing from the bottom to the top, Livingston et al. (2018) observed that freezing in wheat, oats and rye usually began in the oldest leaves and then sequentially to the youngest leaves. Often the youngest tissues (meristems) supercooled to −14 or −16 °C (−18 °C in rye), indicating that younger leaves and meristems can be protected by supercooling (Wisniewski et al. 2014). This strategy would protect meristematic tissues and ensure winter survival (Legge et al. 1983).

The underlying mechanisms of sequential leaf freezing from old to young leaves are unknown and likely involve multiple aspects (Ashworth et al. 1985). For instance, images from high definition IR thermography revealed that freezing in wheat leaves is initiated in xylem vessels (Livingston et al. 2018). Thus, it could be hypothesized that smaller vessels in younger leaves cause a delay in ice propagation (Aloni and Griffith 1991; Liu et al. 2003; Breman et al. 2009) resulting in younger leaves freezing after older leaves. In addition, leaf freezing could be delayed or prevented by cryoprotective molecules such as carbohydrates involved in membrane stabilization or freezing point depression (Dowgert and Steponkus 1984; Hincha et al. 2007) or amino acids acting as non-toxic osmoregulators (Levitt 1980). On the other hand, supercooling is only possible in the absence of intrinsic and extrinsic ice nucleators (Ball et al. 2002; Wisniewski et al. 2008). Since bacterial and fungal populations have their own ice nucleation activity (Ashworth et al. 1985), differences between leaves in the composition of the microbiota could partly explain the capacity of young tissues to supercool.

The purpose of this study was to correlate the sequence of freezing of wheat leaves, observed by IR thermography, to factors that could explain the freezing pattern and account for the greater extent of supercooling of certain tissues. While ultimately this research will elucidate freezing survival mechanisms, we were primarily concerned with the initiation of freezing and not the survival of leaves or plants. For this purpose, a leaf-by-leaf characterization was undertaken of the anatomy of vascular bundles, carbohydrate and amino acid concentrations in leaf sheaths, transcript expression of cryoprotective molecules and the microbiome population, from the oldest external leaf (leaf 1) to the leaf just above the meristem (leaf 6) of whole, cold-acclimated wheat plants.

## Materials and methods

### Plant material

Seeds of the wheat (*Triticum aestivum* L.) cultivar Shirley (North Carolina Foundation seed, Zebulon, NC, USA) were sown into 2.5 cm diameter by 16 cm deep cone-tainers that were filled with Fafard 4P soil mix (Sungro Horticulture, Agawam, MA, USA). They were grown for 4 weeks at 15 °C with a 12 h daylength under 300 μmol m^−2^ s^−1^ of mixed cool-white fluorescent and incandescent lighting. After 4 weeks, they were transferred to a 3 °C cold-acclimating chamber with 12 h of lighting also at 300 µmol m^−2^ s^−1^. Plants were watered daily and fertilized twice weekly with Scotts Miracle-gro complete fertilizer (Scotts Miracle-Grow, Marysville, OH, USA).

**Fig. 1:**
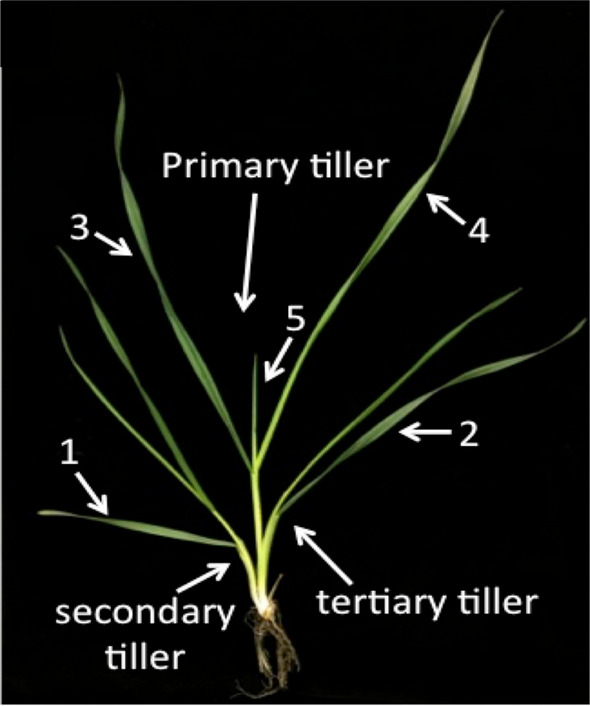
Cold acclimated wheat plant after 8 weeks under controlled growing conditions. Note the 3 tillers at this stage of growth. Also note that the sheath of the first leaf to emerge (labeled “1” which is the oldest leaf and the first to freeze) envelops all 3 tillers. The sheath for leaf 2 envelops the primary and tertiary tillers. Leaves 3, 4, and 5 are on the primary tiller and leaf 6 (the youngest leaf and the last to freeze) is inside leaf 5 and is not visible at this growth stage. (Used with permission, Livingston et al. 2018)

After the 3-week cold-acclimating treatment, plants were at a 4 to 5 leaf stage and had 3 prominent tillers (Fig. 1).

### Leaf freezing assays

#### Leaf section freezing test

Five replicates of four leaves were sampled, with three small leaf segments in sterile microfuge tubes. The tubes were shipped overnight on wet ice to the USDA-ARS Appalachian Fruit Research Station. The small leaf segments, ca. 0.5 × 2.5 cm, were removed with sterile forceps and placed into sterile 1.8 × 12.0 cm tubes with 3 mL of sterile water; all transfers were performed in a sterile laminar flow hood (UV light treatment for 1 h prior to transfer), and sterile kaputs placed on the tubes.

The tubes were inserted approximately 10 cm into a 50% water/ 50% ethylene glycol (v/v) mix contained in a manually adjustable, immersion chiller bath (Forma Scientific Model 2235). Bath temperature was independently monitored via an ethanol thermometer and an Omega model H423 digital thermometer using a thermocouple; the thermocouple was placed into a similar tube as the samples with 3 mL of water. The samples were initially equilibrated at 0 °C, followed by 0.5 °C decrements every 20 min (5 min to achieve the desired temperature and 15 min incubation). The samples were then partially removed from the freezing bath at the end of the incubation and gently swirled by flicking the bottom of the tube, and the tube observed as to whether freezing of the 3 mL occurred. A final temperature of −5.5 °C was used to ensure all samples had frozen.

#### Homogenate droplet freezing test

Five replicates of four leaves were homogenized in 1 mL of sterile water in a microfuge tube and shipped with the leaf segments as described above. The microfuge tubes were briefly spun 10 s at 1000 g to partially settle the debris. A freezing plate (ThermoElectric Cooling America Corp. Model AHP-2700 CPV) was prepared by applying a layer of thermal transfer grease (Fuchs Lubritech Chemplex 1381) on the surface, and strips of parafilm firmly pressed into the grease. Twenty droplets (20 μL) of each replicate/leaf along with equivalent numbers of 20 μL water droplets as a negative control and 20 μL droplets of a water/ice nucleating active (INA) slurry as a positive control were then placed on the parafilm. A thermocouple connected to an Omega model H423 digital thermometer was then taped to the parafilm as an independent temperature measurement. The plate was equilibrated at 0 °C for 15 min followed by 0.5 °C decrements; the plate was allowed to equilibrate at each new temperature for 15 min after achieving the desired temperature. The droplets were then observed at the end of the 15 min equilibration for the phase transition from liquid to solid (apparent as a change from clear to opaque). The percent frozen of each replicate/leaf combination was then recorded at each temperature.

#### Anatomical measurements

Histological protocols were as previously described (Livingston et al. 2009) using Microwave-assisted fixation, dehydration and paraffin embedding of leaf sheaths for leaves 1–6 (Fig. 1). Paraffin-embedded leaves were sectioned at 10 μm and fixed to slides as described (Livingston et al. 2006). After de-paraffinizing with xylene, sections were triple stained with Saffranin, Fast Green and Orange G.

Photographs of the midvein of leaves 1–6 were taken with a Cannon Rebel consumer grade camera mounted on a Nikon Eclipse 50i at 400×. Images were analyzed by Win-cell (Regents Instruments Inc., Quebec, Canada) and the area of xylem vessels within the vessel bundle was quantified. Five replicates of each leaf were quantified for vessel area and analyzed for statistical differences.

#### Biochemical analyses

Roots were trimmed and plants were washed with tap water to remove as much soil from the crowns as possible. Plants were blotted with paper towels to remove excess water.

A bundle of 20–25 plants was placed in the center of a research-grade microwave (Pelco Biowave Pro Tissue Processing System, Ted Pella Inc, Redding, CA, USA) with a temperature probe inserted in the middle of the bundle and microwaved for 2 min at 70 °C and 750 W to prevent enzymatic activity during drying and subsequent analysis (Pelletier et al. 2010). A previous analysis indicated that this was the most accurate tissue preparation protocol for fructan analysis in turfgrass (Bertrand et al. 2011). After microwaving, 1 cm of leaf segment from the base of the crown was collected for leaves 1–5. For leaves 1–4 this segment consisted of the leaf sheath. Leaf sheaths for leaves 5 and 6 were not present at this point of development. Also, since leaf 6 was smaller than 1 cm, the entire leaf was collected. Leaf samples were dried overnight at 70 °C in an oven and shipped to the Quebec Research and Development Centre (Agriculture and Agri-Food Canada) for carbohydrate analysis.

Approximately 200 mg of dried ground leaf samples were incubated for 90 min in tubes with 7 mL of deionized H_2_O at 100 °C to inhibit enzyme activity. Tubes were cooled and left overnight at 4 °C for optimal extraction of soluble carbohydrates. Tubes were then centrifuged for 10 min at 1500 *g*, and 1 mL of the supernatant was collected for the following analyses: soluble carbohydrates (raffinose, sucrose, glucose and fructose); starch; fructans of low degree of polymerisation (LDP 3 to 10), fructans of high degree of polymerisation (HDP > 12) and amino acids.

#### Carbohydrates

Soluble carbohydrates were quantified by HPLC using a Model 515 pump, a Model 717Plus autosampler, and a Model 2410 refractive index detector. Individual carbohydrates were separated on an Aminex HPX-87P (Bio-Rad Laboratories Inc., Hercules, CA, USA) column (300 × 7.8 mm × 9 μm) preceded by a Carbo-P pre-column (Bio-Rad) and eluted isocratically at 80 °C, at a flow rate of 0.5 mL min^−1^, with deionized H_2_O. Carbohydrate concentrations were expressed on a 105 °C dry weight (DW) basis determined using a thermogravimetric analyzer (Model TGA 701, Leco Corporation, St Joseph, MI, USA).

Starch was hydrolyzed by adding 3 mL of digestion buffer (200 mM sodium acetate, pH 4.5) with amyloglucosidase (AGS, 15 U mL^−1^, Sigma-Aldrich) in the tubes used for soluble carbohydrates extraction, containing the remaining supernatant and pellets. Starch standards of known concentrations were included in the hydrolysis batch to establish the linear regression between glucose and starch concentrations. The difference in glucose concentration obtained after and before AGS hydrolysis was fitted into the regression equation to calculate the starch concentration of each sample expressed on a 105 °C DW basis (mg g^−1^ DW).

LDP Fructans (DP 3–10) were analyzed using a Waters ACQUITY Ultra Performance Liquid Chromatography (UPLC) analytical system controlled by the Empower II software (Waters, Milford, MA, USA). Elution was performed at 35 °C with a flow rate of 0.25 mL min^−1^ with the following gradient of eluant A (80% acetonitrile with 0.1% NH_4_OH) and eluant B (30% acetonitrile with 0.1% NH_4_OH): from 0 to 15 min, 70% A and 30% B; from 15 to 17.5 min, 30% A and 70% B, curve 6; from 17.5 to 17.51, 70% A and 30% B, curve 6; from 17.51 to 20 min, stable at 70% A and 30% B. LDP fructans were separated on an ACQUITY UPLC BEH AMIDE 1.7 µm (2.1 × 100 mm) column preceded by a VanGuard (2.1 × 5 mm) pre-column and detected on an Electric Light Scattering Detector (ELSD) set to a gas pressure of 45 psi. The drift tube was set at 50 °C in the cooling mode. Samples were kept at 4 °C in the sample manager throughout the analysis. The degree of polymerization of LDP fructans was established by comparison with elution time of purified standards from Jerusalem artichoke (*Helianthus tuberosus* L.) and the quantity was determined by reference to a fructose standard.

HDP Fructans (DP > 12) were separated on a Shodex KS-804 column preceded by a Shodex KS-G pre-column (Shodex, Tokyo, Japan) eluted isocratically at 50 °C with deionized water at a flow rate of 1.0 mL min^−1^, and were detected on a refractive index detector (Model 2410, Waters). The degree of polymerisation of HDP fructans was estimated by reference to a standard curve established with seven polymaltotriose pullulan standards (Shodex Standard P-82) ranging from a molecular weight of 0.58 × 10^4^ to 85.3 × 10^4^. The retention time on the Shodex column is a function of the log of the molecular weight of pullulan molecules. The concentrations of both HDP and LDP fructans are expressed on an equivalent fructose basis.

#### Amino acids

Twenty-one amino acids were separated and quantified using the Waters ACQUITY UPLC analytical system controlled by the Empower II software (Waters). The amino acids were derivatized using AccQ Tag Ultra reagent (6-aminoquinolyl-N-hydroxysuccinimidyl carbamate). The derivatives were separated on a 2.1 × 100 mm AccQ Tag Ultra column (Waters) and detected with Waters ACQUITY Tunable UV detector at 260 nm under the chromatographic conditions described in Cohen (2000). Peak identity and amino acid quantity were determined by comparison to a standard mix containing the 21 amino acids. Results from amino acid determination were expressed as concentrations on DW basis (µmol g^−1^ DW).

### Microbiome analyses

#### Amplicon-based sequencing of the leaf microbiome

Leaves of young wheat plants were sampled for analysis of the fungal and bacterial microbiome. Leaves of sequential age were sampled, ranging from oldest (leaf 1) to youngest (leaf 6). Pools of leaves representing six biological replicates were collected at the same time, frozen in liquid nitrogen, and then processed for amplicon-sequencing of fungal and bacterial taxa. The protocol utilized for the sequencing was as described in detail in Abdelfattah et al. (2020) with the exception of the primer set used to amplify the fungal ITS region (described below).

DNA was extracted from the leaf samples using a DNeasy PowerLyzer PowerSoil Kit (Qiagen, Germantown, MD, USA). Initial tissue disruption of 250 mg was performed with a Qiagen PowerLyzer 24 Homogenizer. DNA extractions were automated using a Qiagen QiaCube, using the processing routine recommended by the manufacturer for the PowerSoil kit. Extracted DNA was used as the template for amplicon PCR reactions that amplified the bacterial 16S ribosomal region and the fungal internal transcribed spacer (ITS) region. ITS amplicons were amplified using the universal primers 5F and 16R. 16S amplicons were amplified using the universal primers 515F and 806R in conjunction with peptide nucleic acids (PNAs) (PNA Bio) added to inhibit amplification of ribosomal and mitochondrial sequences. ITS amplicons were amplified using the universal primer set 5F and 86R. All primers were modified to include the necessary Illumina adapters (www.illumina.com) for subsequent PCR addition of Illumina indexes for multiplexing.

For bacterial (16S) amplicon generation, PCR reactions were conducted in a total volume of 25 μL containing 12.5 μL of KAPA HiFi HotStart ReadyMix (Kapa Biosystems, Sigma-Aldrich), 1.0 μL of each primer (10 μM), 2.5 μL of mitochondrial PNA (5 μM), 2.5 μL of plastid PNA (5 μM), 2.5 μL of DNA template, and 3 μL nuclease-free water. Reactions were incubated in a T100 thermal cycler (BioRad) at 98 °C for 5 min followed by 30 cycles of 95 °C for 30 s, 78 °C for 5 s, 55 °C for 30 s, 72 °C for 30 s and concluding with a final extension at 72 °C for 5 min. For fungal (ITS) amplicon generation, 25 μL PCR reactions contained 12.5 μL of KAPA HiFi HotStart ReadyMix (Sigma-Aldrich), 1.0 μL of each primer (10 μM), 1.0 μL of blocking oligo (10 μM), 2.5 μL of DNA template, and 7 μL nuclease-free water. Reactions were incubated in a T100 thermal cycler (BioRad) at 95 °C for 5 min followed by 30 cycles of 95 °C for 30 s, 55 °C for 30 s, 72 °C for 30 s and concluding with a final extension at 72 °C for 5 min. Library preparation following amplicon PCR was performed as specified in the Illumina 16s Metagenomic Sequencing Library Preparation guide precisely as outlined in conjunction with the use of a Nextera Index Kit (Illumina Inc, San Diego, CA, USA) containing 96 indexes. Subsequent library size, quality, and confirmation of the absence of adapter dimers was performed on an Agilent 2100 Bioanalyzer (Agilent, Santa Clara, CA, USA). Paired-end sequencing of amplicons was done on an Illumina MiSeq (Illumina) sequencer with a V3 600-cycle Reagent Kit (Illumina).

Qiime2 (Bolyen et al. 2019) was used for demultiplexing, merging, quality trimming of reads, acquired sequence variant (ASV) table generation, and rarefaction to account for uneven sequencing depth. Taxonomic clustering ASVs was done using a similarity threshold of 97% against the GreenGenes (DeSantis et al. 2006) database for 16S reads and against the UNITE (Abarenkov et al. 2010) database for ITS reads. MetagenomeSeq’s Cumulative Sum Scaling (CSS) (Paulson et al. 2013) was used as a normalization method subsequent to community composition analyses, including the calculation of Bray–Curtis dissimilarity metrics (Bray and Curtis 1957), the construction of PCoA plots, and PERMANOVA analyses.

#### RNA isolation and ice binding protein gene analysis

Cold-acclimated leaves 1–6 were collected from 8 plants and crowns were collected from 3 plants. Non acclimated samples were harvested from plants that were at approximately the same growth stage as cold-acclimated plants and then frozen and ground in liquid nitrogen. Total RNA was isolated by following the instructions of RNeasy Plant Mini Kit (Qiagen) and quantified using a NanoDrop ND-1000 spectrophotometer (NanoDrop Technologies, Wilmington, DE, USA). cDNA was synthesized from 1 mg of total RNA with the use of iScript Reverse Transcription Supermix (Bio-Rad). Primers of TaIRI-1-forward (5′ TCTGGGAGCAACCATGTCGT 3′), TaIRI-1-reverse (5′ CACCCAAGTGATTGTGGGCTA 3′), TaCHT-1-forward (5′ ACACCCAGAGGAACTTCGCTA 3′), TaCHT-1-reverse (5′ TCCTTTATTCCACGACGGCAT 3′), Ubiquitin (UBI)-forward (5′ CCTTGGCGGACTACAACATC 3′), and UBI-reverse (5′ GCAACGACAGACACAGACC 3′) were designed based on 3′UTR by Primer-BLAST (https://www.ncbi.nlm.nih.gov/tools/primer-blast/index.cgi). Five micro-liters of SsoAdvanced Universal SYBR (Bio-Rad) were mixed with an equal volume of mixture of 250 nM of forward and reverse primer and 100 mg of cDNA temperate. Real-time PCR was performed using CFX96 Touch Real-Time PCR Detection System (Bio-Rad) with a program of 95 °C for 30 s, 45 cycles of 95 °C for 15 s, and 60 °C for 30 s. The comparative threshold cycle (*C*_T_) method was used to determine the relative amount of gene expression, with the expression of UBI used as an internal control. Mean values were calculated from three independent experiments. Three biological repeats were analyzed.

## Results and discussion

Ice formation within plant tissue is arguably the most important aspect of freezing stress. The temperature at which ice is initiated in plants is not only affected by environmental conditions such as the rate of temperature change, temperature nadir and plant-associated microbiota (Bohnert et al. 1995; Wisniewski et al. 2009, 2014; Dumans and Wisniewski 2014), but also by characteristics of the plant tissue per se.

A previous study using IR thermography demonstrated that freezing in wheat occurs in two stages with a third stage that was not visible in IR but was implied, since plants fully recovered after stages 1 and 2 but died if the temperature was reduced beyond the second stage (Livingston et al. 2018). IR thermography also revealed that freezing begins within the vascular system in the leaf that was the first to emerge (leaf 1, the oldest leaf) and progresses sequentially to the last leaf to emerge (leaf 6, the youngest leaf) as temperatures declined (Livingston et al. 2018). This freezing pattern was observed in wheat grown under both natural as well as controlled conditions and in plants that were in a vegetative as well as reproductive stage of growth (Livingston et al. 2018) as well as in several other species of plants (Kaku and Salt 1968; Pearce and Fuller 2001; Hacker and Neuner 2007).

The goal of the present study was to identify anatomical, metabolic and microbial factors that contribute to the sequential freezing in wheat leaves to ultimately protect young meristematic tissues by supercooling.

### Ice nucleation activity

**Fig. 2:**
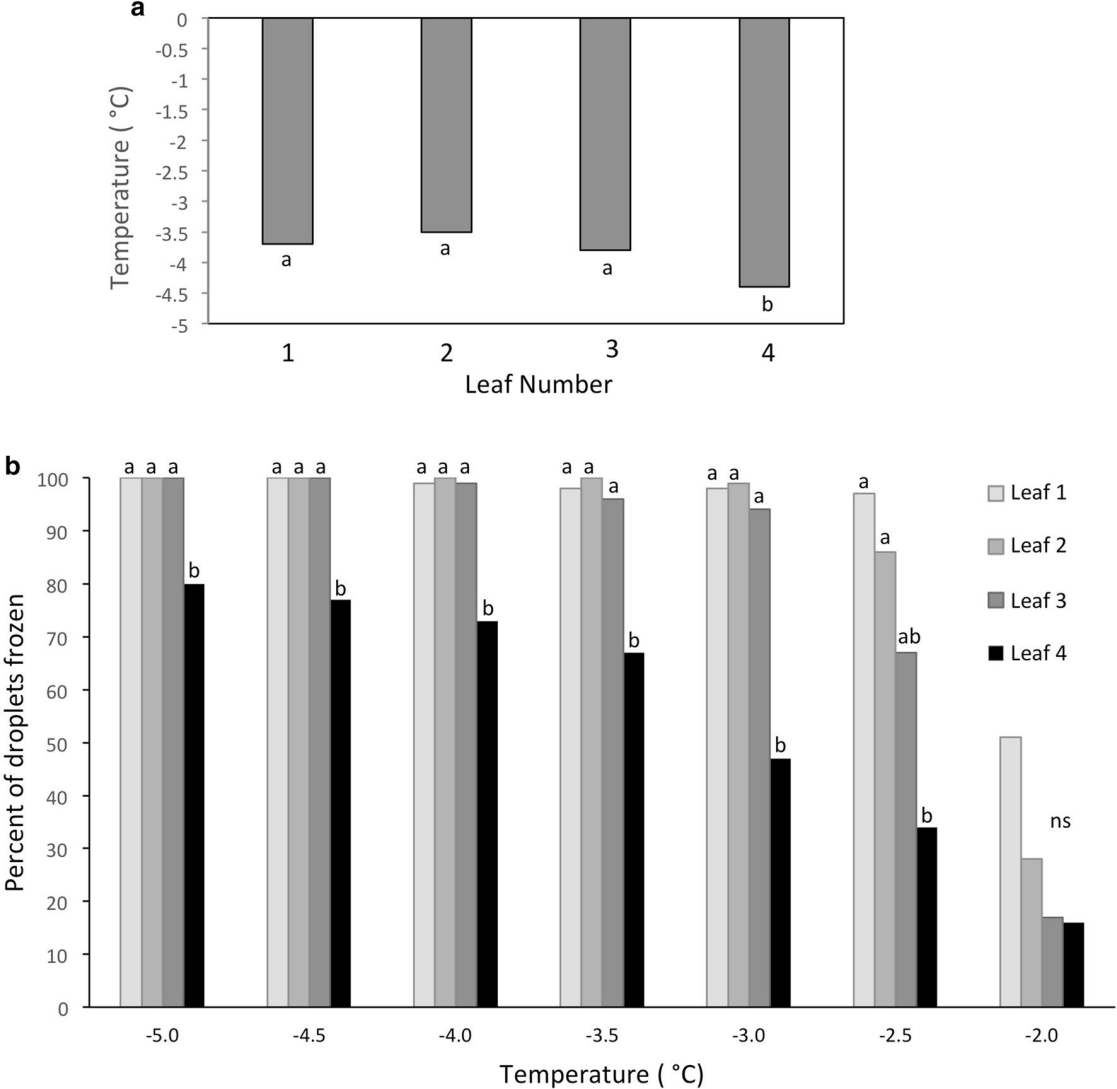
Age-related freezing activity in wheat leaves. **a** Leaf freezing-bath assay. Average nucleation temperature of water in test tubes containing segments of wheat leaves. Leaf 1—was the oldest leaf, Leaves 2–4, sequentially younger leaves. Letters below the columns that are different indicate a significant (*P* = 0.05, *n* = 5) difference between leaves as determined by Tukeys’ HSD analysis. **b** Ice-nucleation activity in wheat leaf homogenates as determined by a drop freezing assay. Droplets used in the analysis represent homogenates of sequentially older leaves. Letters above columns at each temperature that are different indicate a significant (*P* = 0.05, *n* = 20) difference between leaves as determined by Pairwise Multiple Comparison Analysis (Holm–Sidak method). Note that no droplets froze between −1.5 and 0 °C and are, therefore, not shown

The ability of leaf segments and leaf homogenates to induce freezing was examined to confirm the age-related freezing of wheat leaves observed by infrared thermography. Although the results exhibited a high level of variability, a clear propensity for younger leaves to freeze at a lower temperature than older leaves was observed (Fig. 2a). This was especially evident for leaf 4 (the youngest leaf tested). Younger leaves (5 and 6) could not be evaluated with this method, because it was not possible to obtain a sufficient number of intact leaves due to their small size. Importantly, the leaf mass for each age leaf was adjusted to be approximately equal so that leaf size was not a factor in the freezing response. Although, the number of extrinsic nucleating agents, such as Ice Nucleation Active (INA) bacteria, would still have been much reduced based on the number of ASVs retrieved from the younger leaves. This aspect is discussed in detail in a later portion of the manuscript.

A freezing drop assay was also conducted using leaf homogenates (Fig. 2b). The results were similar to those obtained in the leaf segment freezing bath assay. A notable decrease in the percentage of frozen drops was evident for the homogenate of leaf 4. While nearly all, or all of the homogenate droplets of leaves 1–3 had frozen at −3 and −4 °C, respectively, only 70–75% of the homogenate droplets of leaf 4 had frozen. These results support the observations made with infrared thermography on intact plants under controlled and natural conditions. They also indicate that intrinsic or extrinsic factors contribute to determining the temperature at which wheat leaves will freeze and the temperature at which freezing is initiated. Since freezing potential is a stochastic event, our data confirms that the probability of a freezing event occurring at a particular temperature is reduced in younger leaves.

### Anatomy

Liu et al (2003) discussed “capillary freezing point depression” and demonstrated that capillaries of a smaller diameter supercooled to a greater extent than larger diameter capillaries. This implies that plants with smaller diameter xylem vessels freeze at a lower temperature. Breman et al. (2009) found that bahiagrass cultivars with smaller vessel diameters were in fact more freezing tolerant than those with larger xylem vessels, presumably, because vital tissues in plants with smaller xylem vessels supercooled to a greater extent. Kuprian et al. (2016) described a delay in freezing into flower buds of *Calluna vulgaris* and provided evidence that the diameter of vessels leading into buds was at least partly responsible for the delay in inoculation of buds. Ashworth and Abeles (1984) and Schulte (2003) describe similar examples of vessel diameter affecting ice propagation in plants.

**Fig. 3:**
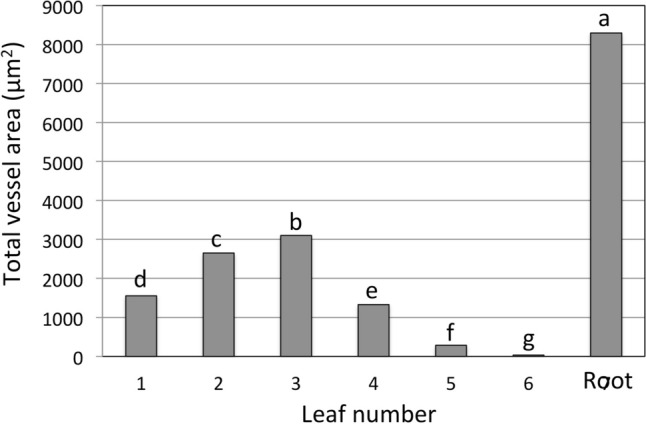
Average area of xylem vessels within the midrib bundle sheath of leaves and in the stele of roots. Leaf 1 is the oldest leaf (the first to emerge) and the first leaf to freeze on intact plants. Although the root, shown here, was always the first tissue of intact plants to freeze. Leaf 6 is the youngest leaf (the last to emerge) and froze last or in some cases did not freeze. Columns with the same letter are not significantly different at *P* = 0.05, *n* = 30

To see if these findings might explain sequential freezing in wheat we measured the average area of xylem vessels within the midrib bundle sheath of leaves and in the stele of roots (Fig. 3). We observed that the cross sectional area of vessels in the wheat roots was very significantly larger (Fig. 3) than any vessel area in leaves and might explain why freezing began in roots. After breaching the root/shoot interface and entering the crown, the age-related freezing sequence of leaves began. Breman et al. (2009) reported that older leaves had larger vessel diameters relative to younger leaves. While the vessel area of the midrib in leaves 3, 4, 5, and 6 was correlated to age (3 = old, 6 = young), as well as freezing sequence, the area of vessels in leaves 1 and 2 did not correlate to leaf age (Fig. 3).

**Fig. 4:**
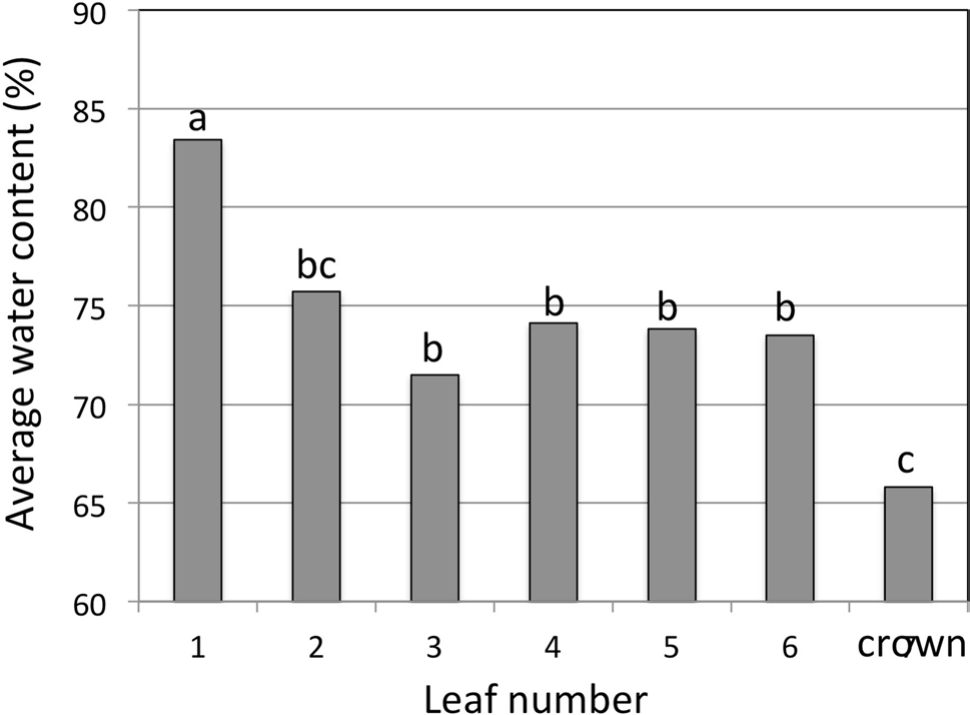
Average water content in leaf sheaths of wheat leaves and crowns. Leaf 1 is the oldest leaf (the first to emerge) and the first leaf to freeze on intact plants. Leaf 6 is the youngest leaf (the last to emerge) and froze last or in some cases did not freeze. All the leaves are attached to the crown from which the vascular system for each leaf emerges. Columns with the same letter are not significantly different at *P* = 0.05, *n* = 13

Thus it seems that vessel diameter could contribute to the delay of freezing of younger leaves, while it is possible that freezing was initiated in leaves 1 and 2 for a reason other than vessel diameter. For example, a high water content has been associated with the initiation of freezing at warmer temperatures (Levitt 1980; Olien and Smith 1981). Since the water content of leaves 1 and 2 was higher than the other leaves, this may explain why leaves 1and 2 froze first (Fig. 4). Freezing in leaves 3, 4, 5, and 6, however, was not correlated to water content.

It is also possible that our vessel area measurements were not made at the precise point where ice nucleation began in each leaf sheath. Kuprian et al. (2016) related diameter of pit membranes at the junction of branches and buds to freezing point depression of *C. vulgaris* buds. A constriction of vessels at the root–shoot junction in rye, wheat and triticale reduced the rate of ice propagation from roots into crowns (Aloni and Griffith 1991; Zamecnik et al. 1994). While wheat leaves join the crown at different positions it was difficult to determine the precise point of attachment for each leaf in this study. The tissue at the crown-leaf junction region is very diverse and it would be difficult if not impossible to determine the precise vessel through which ice propagation from the crown to leaf was being controlled.

Three-dimensional reconstruction (not shown) of the vascular system in wheat crowns demonstrated that leaves 1 and 2 are joined to the crown at the base, just above the root–shoot junction. Leaves 3 and 4 join the crown further up the sides of the crown and leaves 5 and 6 protrude upwards at the top of the crown. It is possible that as ice forms at the base of the crown the ice front simply moves upwards within the crown and inoculates the vascular system of each leaf junction as it is encountered.

Leegood (2008) states that different vessel bundles in grass leaves have different functions with some conducting primarily solute efflux and water influx, while others may engage in the inverse. It seems likely that different leaves on the same plant might have similar differences in function depending on growth stage; this could affect the means by which freezing is initiated. It is possible that vessel diameter was the primary mechanism of freezing point depression in leaves 3, 4, 5 and 6 but some other mechanism determined ice nucleation in leaves 1 and 2. Additional factors likely related to why leaves 1 and 2 froze first and why leaves 3, 4, 5 and 6 were not correlated to water content are discussed below.

### Cryoprotective compounds

#### Carbohydrates

**Fig. 5:**
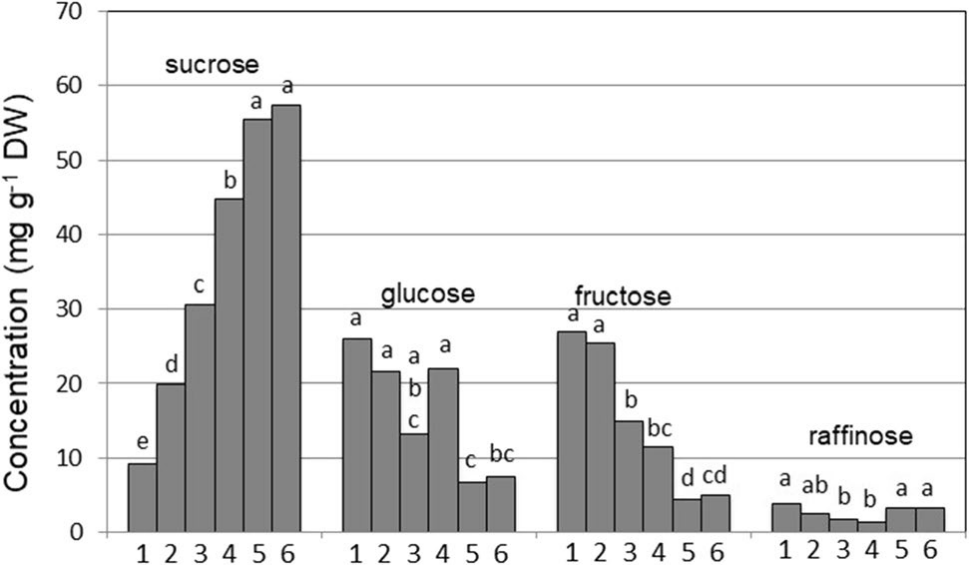
Differences in concentrations on a dry weight basis (DW) of sucrose, glucose fructose and raffinose between six wheat leaves of chronological age from the oldest (leaf 1—the first leaf to freeze) to the youngest meristematic leaf 6. For each sugar, different letters indicate significant differences between the leaves, as determined by Fisher’s least significant difference (LSD) at *P* = 0.05, *n* = 5

**Fig. 6:**
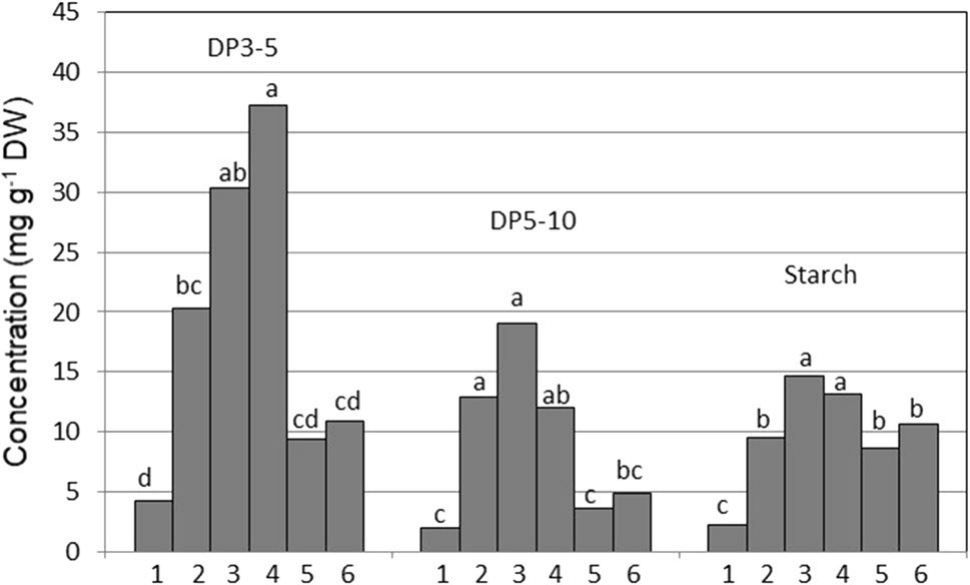
Differences in concentrations of two classes of fructans of low polymerization degree (DP3 to 5 had a polymerization range between 3 and 5, while DP5 to 10 had a polymerization range between 5 and 10), and differences in starch concentration between six wheat leaves of chronological age from the oldest (leaf 1, the first leaf to freeze) to the youngest meristematic leaf 6. For each carbohydrate, different letters indicate significant differences between the leaves, as determined by the Fisher’s least significant difference (LSD) test at *P* = 0.05, *n* = 5

**Fig. 7:**
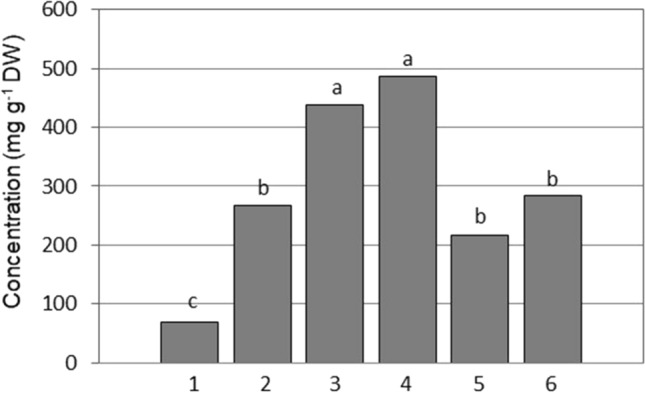
Differences in concentrations of fructans of high degree of polymerization (DP > 12) between six wheat leaves of chronological age from the oldest leaf 1 (first to freeze) to the youngest meristematic leaf 6. Different letters indicate significant differences between the leaves, as determined by the Fisher’s least significant difference (LSD) test at *P* = 0.05, *n* = 5

Besides anatomical characteristics, compounds that depress the freezing point of a given tissue could promote supercooling. To investigate this possibility, an analysis of cold-associated carbohydrates in separate leaves revealed significant differences in concentrations between leaves (Figs. 5, 6 and 7).

Sucrose concentration increased significantly from 10 mg g^−1^ dry weight (DW) in the oldest leaves to 60 mg g^−1^ DW in the youngest leaves, which was correlated with the pattern of leaf freezing (Fig. 5). Sucrose has been shown to dramatically reduce the rate of ice migration and to decrease nucleation temperature in canola (Gusta et al. 2004) and oat leaves (Livingston 2007) presumably due to the ability of solutes to reduce the freezing point colligatively. Besides reducing the freezing point, sucrose directly protects cell membranes by interacting with the phosphate in their lipid phosphate headgroups, thus increasing membrane viscosity and stability through a vitrification state (Strauss and Hauser 1986). The trisaccharide raffinose, in the raffinose family of oligosaccharides (RFOs), had a maximum concentration in younger leaves 5 and 6 (Fig. 6). Raffinose plays a role similar to sucrose in stabilizing membranes but, due to its subcellular localization, its protective action may be restricted to chloroplast inner membranes (Nägele and Heyer 2013). Even though our aim here was to relate carbohydrates to freezing sequence rather than freezing tolerance, it is possible that besides a freeze-protective role, raffinose could have a supercooling effect similar to sucrose. In contrast to raffinose, glucose and fructose concentrations were lower in younger than in older leaves, likely involving a decreased activity of sucrose synthase in young tissues.

In addition to the specific role of individual sugars, the effects of different sugar types on membrane protection could be compared on a sugar ring basis. One of the well-known effects of sugars is that they can prevent the dehydration-induced increase in the temperature at which membranes undergo the gel–fluid phase transition. The mechanisms for this can be understood in terms of the effects of solutes on hydration forces between membranes (Koster et al. 2000). The result of a quantitative study of the effect of sugars types on the membrane gel–fluid phase transition shows that the maximum effect occurs at around 1.5 sugar rings per membrane phospholipid molecules (Lenné et al. 2007). We observed a gradual increase in sugar ring concentration from leaf 1 to leaf 6 (Table 1). Although the sugar ring:membrane lipid ratio has not been measured in our experiment, we might posit the hypothesis that the increase in sugar ring concentration, from 82 in leaf 1 to 136 in leaf 6, increased the sugar ring:membrane lipid ratio from a suboptimal value in leaf 1 to a ratio approaching the maximal protecting effects in leaf 6. This may partially explain the effect on freezing tolerance of each leaf, since we normally observe that older leaves are not as freezing tolerant as younger leaves (unpublished data). But, whether the sugar ring:membrane lipid ratio differences between leaves would contribute to the sequential freezing previously observed (Livingston et al. 2018) is unknown.

**Table 1:** Total sugar ring concentration calculated as 2 × sucrose + 1 × glucose + 1 × fructose + 3 × raffinose, in six wheat leaves of chronological age from the oldest leaf 1 to the youngest meristematic leaf 6

Leaf number	Number of rings
1	83c
2	94bc
3	94bc
4	127ab
5	132a
6	137a

Different letters indicate significant differences between the leaves, as determined by the Fisher’s least significant difference (LSD) test at *P* = 0.05, *n* = 5

Fructan is another class of carbohydrates involved in the protection of membranes (Hincha et al. 2007; Livingston et al. 2009) during freezing. However, as in the case of raffinose, a protective role of fructan once ice has formed in tissues likely does not explain the sequential supercooling of water in leaves. While sucrose and the sequence of leaf freezing were correlated, the concentrations of fructans of low DP (DP 3–5) (Fig. 6) and high DP (DP > 12) (Fig. 7) were only related to the sequence of freezing of leaves 1–4 and not of younger leaves 5 and 6 suggesting that different mechanisms could be at play in the supercooling of younger leaves. Leegood (2008) discusses the transition of leaves from sink leaves to source leaves as plants grow older which allows speculation that at the growth stage we selected, leaves 5 and 6 might be either still in a sink phase or at least transitioning to a source phase. Therefore, while carbohydrate concentrations could explain the sequence of freezing in older leaves, the propagation of ice into very young leaves could be regulated by anatomical barriers such as narrow xylem vessels that would ostensibly result in capillary freezing point depression (Livingston et al. 2018).

High degree of polymerization (HDP) fructans were markedly abundant in leaf 4, where their concentration reached 500 mg g^−1^ DW, and was the most abundant molecule in leaf 4 (Fig. 7). Fructans are primarily localized in the vacuole, suggesting that their contribution to membrane stabilization may be restricted to the tonoplast. The detection of fructan in the apoplast of plants exposed to freeze acclimation (Olien 1984; Livingston and Henson 1998) also suggests a role in the protection of the plasma membrane, where they can be delivered by a vesicle-mediated transport (Valluru et al. 2008). Fructans have the capacity to stabilize membranes during drying by inserting at least part of the polysaccharide into the lipid head-group region of the membrane (Hincha et al. 2007; Livingston et al. 2009). Whether these freeze-protective mechanisms can also explain a sequential supercoiling of separate leaves is not known.

#### Amino acids

**Fig. 8:**
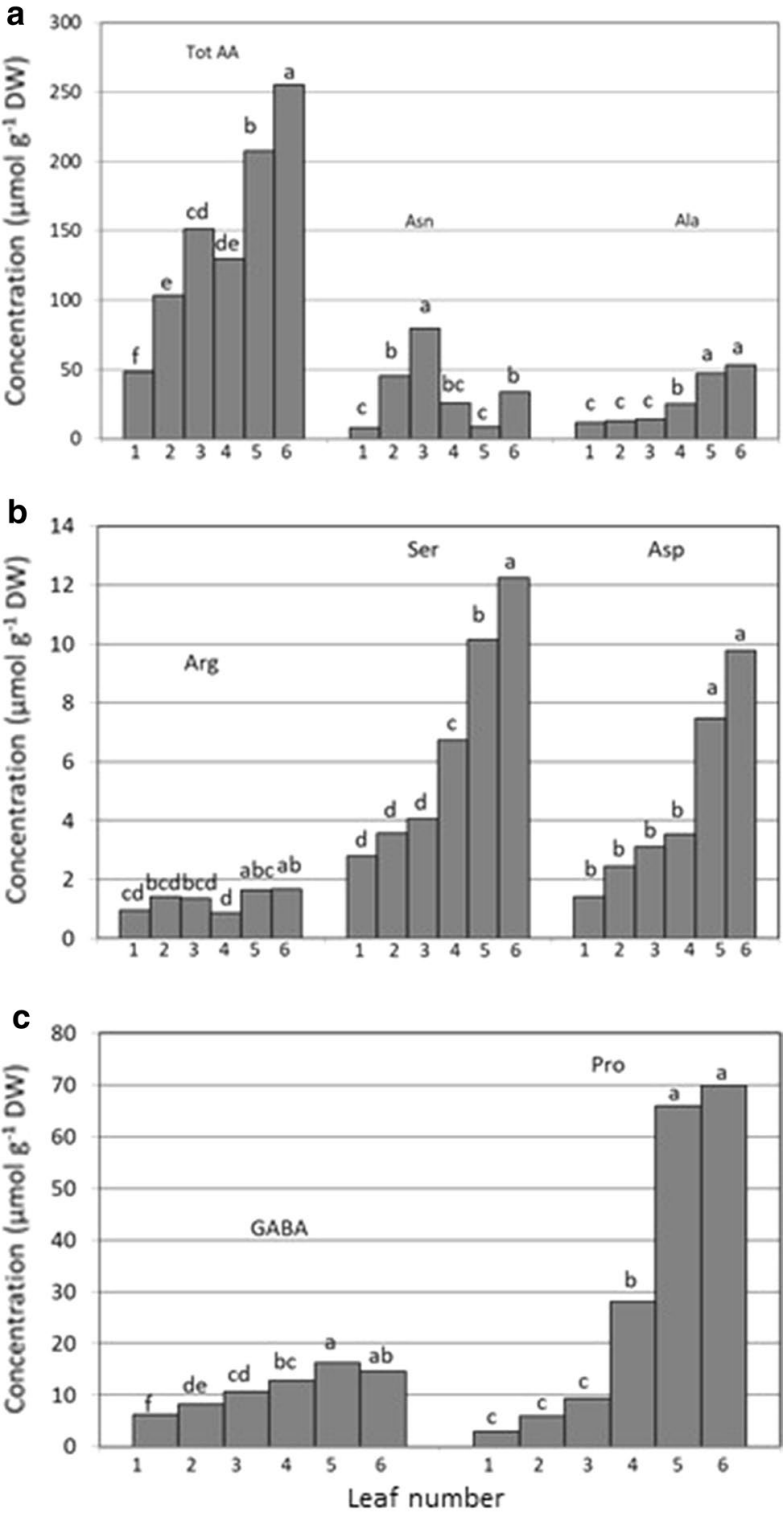
Differences in concentrations of total free amino acids (Tot AA, sum of 21 amino acids). **a** Asparagine (Asn) and alanine (Ala), **b** arginine (Arg), serine (Ser), and aspartic acid (Asp), **c** gamma-amino butyric acid (GABA), and proline (Pro) between six wheat leaves of chronological age from the oldest (leaf 1—the first leaf to freeze) to the youngest meristematic leaf 6. For each amino acid, different letters indicate significant differences between the leaves, as determined by Fisher’s least significant difference (LSD) at *P* =  0.05, *n* = 3

The concentration of total and of several individual amino acids differed significantly between leaves of different chronological age (Fig. 8a–c). The type of amino acids that accumulated in leaves showed a marked transition between leaves 3 and 4. In older leaves the concentration of asparagine (Fig. 8b), which is mostly involved in nitrogen assimilation transport and storage, was correlated with the sequence of freezing. Then, a switch from asparagine to proline occurs and proline becomes the most abundant amino acid (Fig. 8c). The asparagine/proline switch could have been made via the turnover of serine and aspartic acids (Fig. 8b) that have been reported to serve as carbon skeleton for amino acids biosynthesis (Hildebrandt et al. 2015).

Research has demonstrated that proline levels undergo significant fluctuation in response to environmental stress (Bohnert et al. 1995). In this study a positive relationship between proline concentration and the sequence of leaves freezing was demonstrated (Fig. 8c) suggesting that proline could at least partially explain a depression of the freezing point of leaves. In fact, the addition of proline in freezing medium has been shown to increase the cryopreservation of animal (Li et al. 2003), plant (Brison et al. 1995) and algal cells (Kuwano et al. 2004). While the precise mode of action of proline remains largely a matter of speculation, various cryoprotective roles have been attributed to proline. Poustini et al. (2007) found a positive correlation between proline concentration and osmotic potential, and concluded that proline contributes to the osmotic adjustment in wheat under water stress without interfering with normal cellular processes and biochemical reactions. Proline molecules can intercalate between the head groups of membrane phospholipids during freeze dehydration. This helps to reduce mechanical stresses in the membranes, or alter the physical properties of membranes making them less prone to a liquid crystalline-to-gel transition (Hoekstra et al. 2001). It has also been suggested that proline molecules can directly replace missing water molecules between the phospholipids headgroups (Rudolph et al. 1986). However, as stated for fructan, it is not known whether these freeze-protective mechanisms can also explain a sequential supercoiling of separate leaves.

### Microbiome

#### Microbial composition of leaves

The role of extrinsic and intrinsic factors responsible for inducing freezing in plants have been investigated for many years. In this regard, numerous taxa of bacteria and some fungi have been demonstrated to have a high level of ice nucleation activity (Ashworth and Kieft 1995). The presence of an extrinsic, ice-nucleating agent, whether of biological or mineral origin, would initiate freezing events at warmer sub-zero temperatures in plant tissues than would occur in their absence, i.e., overriding the innate ability of the plant tissues to supercool prior to freezing. Therefore, amplicon-based high-throughput sequencing was used to provide information on the number of ASVs that were present on leaves, the number of unique ASVs on the different aged leaves, and the bacterial and fungal taxa that were present.

#### Bacterial ASVs

**Fig. 9:**
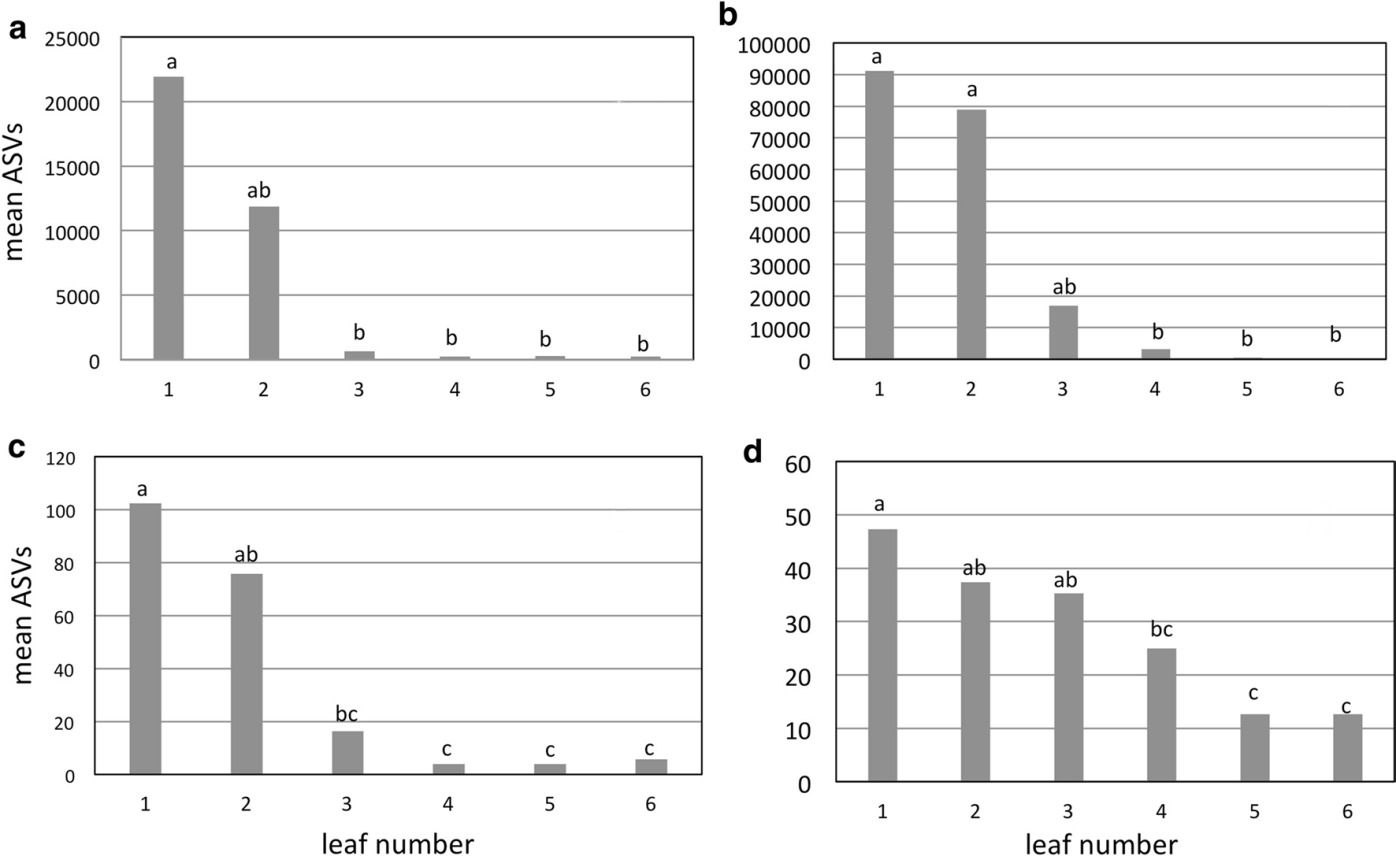
Mean number of total bacterial (**a**), total fungal (**b**), unique bacterial (**c**) and unique fugal (**d**) acquired sequence variants (ASVs) found in sequentially younger leaves of wheat. Leaf 1 is the oldest leaf (the first to emerge) and the first leaf to freeze on intact plants. Leaf 6 is the youngest leaf (the last to emerge) and froze last or in some cases did not freeze. Columns with the same letter are not significantly different at *P* = 0.05, *n* = 4

**Fig. 10:**
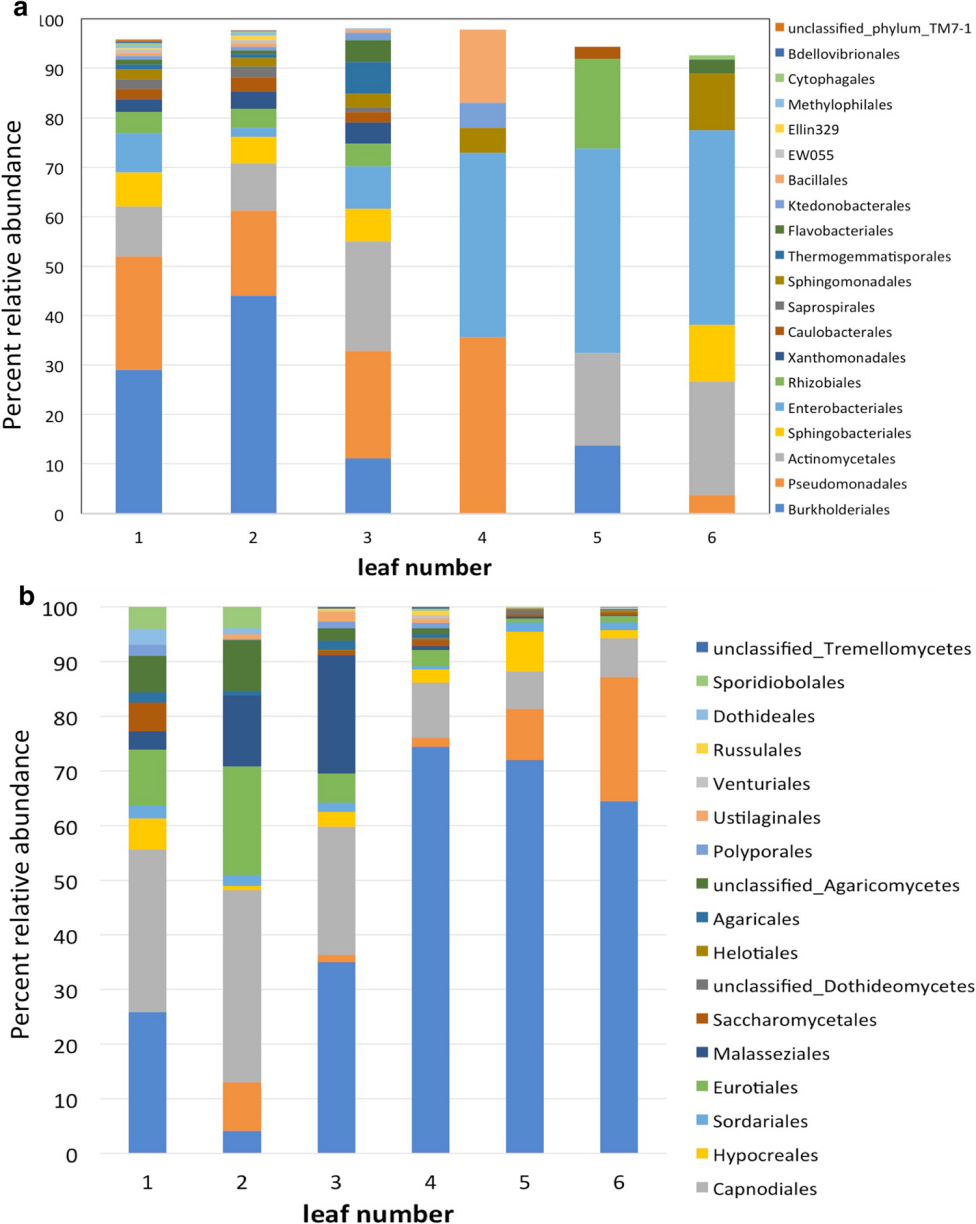
Bar plot showing the leaf age-related relative abundance of bacterial (**a**) and fungal phyla (**b**) in wheat leaves numbered 1 (oldest and the first leaf to freeze) to 6 (youngest). Listed taxa represent the orders of bacteria and fungi present representing > 0.05% of the determined ASVs

The population level of bacteria on leaves clearly decreased from older to younger leaves (Fig. 9a). Although an average of over 20,000 ASVs was observed in the oldest leaf (leaf 1), the average number of ASVs in younger leaves decreased rapidly, especially after leaf 2. Whereas the average number of ASVs found in leaf 3 was approximately 900 ASVs, leaves 4–6 possessed an average of 1–200 ASVs. A similar trend was observed for the number of unique ASVs (Fig. 9c). Amplicon-based taxonomic assessment of the bacterial taxa indicated that the three most abundant orders were Burkholderiales, Pseudomonales, and Enterobacteriales (Fig. 10a). Taxonomic assignment below the family level based on amplicon-sequencing of 16s using universal primer sets can be problematic. Homology-based alignment of the sequences, however, indicated the presence of both *Pseudomonas spp*. and *Pseudomonas viridiflava*. Notably, Pseudomonads, especially *Pseudomonas syringae*, are known to exhibit ice-nucleation activity. Notably, the relative abundance of *Pseudomonas* spp. was greatest in the oldest leaves, which were the first to freeze, and then decreased in the younger leaves. This was especially noticeable in leaves 4–6, where their presence was virtually absent, corresponding with the decrease in ice-nucleation activity in leaf 4 observed in the freezing assays (Fig. 2a, b).

#### Fungal ASVs

The total number of fungal ASVs (Fig. 9b) and number of unique ASVs (Fig. 9d) was similar to the results obtained for bacterial ASVs (Fig. 9a, c). A clear decrease in abundance was observed from older to younger leaves. The average total number of ASVs in the oldest leave was approximately 90,000 and only a few hundred in the youngest leaves (Fig. 9b). A consistent and sharp decrease was evident beginning in leaf 3 among leaves 1 (oldest leaf and the first to freeze) to 6 (youngest leaf and the last to freeze). A similar age-related decrease was observed in the number of unique ASVs (Fig. 9d). Amplicon-based taxonomic assessment of the fungal taxa indicated that the three most abundant orders were Polyporales, Ustilaginales, and Venturiales (Fig. 10b). No further taxonomic identification was pursued. Several species of fungi have also been reported to exhibit ice-nucleation activity, especially in the genus, *Fusarium* (Ashworth and Kieft 1995). In some cases, however, the ice-nucleation activity in fungi has been associated with contamination of spores with *Pseudomonas syringae* (Morris et al. 2013).

#### Transcript expression of ice binding proteins

Although this study does not address the difference in freezing tolerance between leaves, it could be posited that ice recrystallization inhibition plays a part in initiation of freezing and help to explain the freezing sequence we observed. We thus assessed the transcript levels coding for two Ice-binding proteins (IBPs) also called antifreeze proteins: the wheat ice recrystallization inhibition proteins (*TaIRI*) and *Triticum aestivum* chitinase-1 (*TaCHT-1*). We observed that the expression of *TaIRI-1* and *TaCHT-1* transcripts were very low in non-acclimated tissues (data not shown). However, after cold acclimation, *TaIRI-1* was induced 19, 16, 12, 1.2, 1.8, and twofold in leaf 1–6, respectively, while *TaCHT-1* was induced in these six leaves 43, 71, 2, 1, 1, and onefold, respectively (unpublished results).

Ice-binding proteins allow many organisms to thrive in normally injurious freezing conditions (Dolev et al. 2016) by controlling the growth of ice crystals (Griffith et al. 2005; Bredow and Walker 2017). However, it has been determined that IBP's in plants only lower the freezing point of liquid water by a fraction of a degree (Tremblay et al. 2005) so this parameter is likely not a significant explanation for the sequence of freezing we observed.

In wheat, ice recrystallization is inhibited by the expression of specific protein cryoprotectants (Yeh et al. 2000; Kontogiorgos et al. 2007). The wheat ice recrystallization inhibition proteins (*TaIRI*) and chitinases (CHTs) exhibit ice recrystallization inhibition activity, prior to actual freezing in different organs. In addition, their transcripts are induced during cold acclimation and cold shock conditions (Ergon et al. 1998; Yeh et al. 2000; Gaudet et al. 2003; Tremblay et al. 2005; Nakamura et al. 2008; Winfield et al. 2010). The accumulation of transcripts encoding cryoprotective proteins during cold acclimation suggests a prophylactic mechanism prior to actual freezing.

Because co-occurring stresses are tied to various pathogens and freezing events, some IBPs that belong to the thaumatin-like, chitinase-like, and glucanase-like proteins are also known as pathogenesis-related proteins in winter cereals (Yu and Griffith 2001; Singh et al. 2007). Interestingly, the expression levels of *TaIRI-1* and *TaCHT-1* within leaves also positively correlated with the abundance and complexity of bacterial and fungal communities, suggesting *TaIRI* and *TaCHT* proteins may prevent freezing associated with pathogenic ice nucleation (Wu et al. 2009).

## Conclusion

Understanding the mechanisms used by plants to survive freezing and protect their meristematic tissues is key to understanding plant survival and worldwide repartition in colder regions. The results presented here indicate that several mechanisms could explain the observation that leaves of wheat (and possibly other grasses) freeze in an age-dependent sequence with older leaves freezing first. In fact, it might be argued that one does not have to investigate multiple species of plants to understand freezing tolerance in plants; one could gain an extensive understanding of freezing tolerance simply by studying various tissues within the same plant.

The deployment of multiple mechanisms likely protects meristematic tissues at the lowest energy cost for the plant. While vessel diameter and water content were correlated to freezing order in some leaves, in others they were not. Several carbohydrates, most notably sucrose, were also correlated with freezing order but fructans were only correlated to freezing in older leaves. Amino acids, particularly proline, could also partially explain freezing order. Pseudomonads have been shown to induce freezing and the results in this study suggest that they, among others, might explain the sequence of freezing, since their access seems to be denied to younger leaves and meristems by mechanisms that remain to be discovered. In older leaves (leaves 1–3) bacteria and fungi that were correlated to freezing order could help limit nucleation of freezing and explain the order of freezing there. The identification of anatomical features and compounds or microbes involved in promoting supercooling could be used as selection parameters in breeding programs and in crop management to reduce freeze damage.

### Author contribution statement

DL Concieved of the study, performed freeze-tests, anatomical analysis and coordinated writing and editing of the mss. AB Conducted carbohydrate and amino acid analysis, and interpreted results. MW Concieved of the homogenet droplet test and microbiome analysis; performed the microbiome analysis and interpreted results. RT Performed RNA assays and interpreted results. TT Provided plant care, performed histological analysis and helped interpret results. LG Helped interpret results and wrote sections of the mss. TA Performed homogenous droplet analysis and helped interpret results.

## Abbreviations

ASVs: Acquired sequence variants
LDP: Low degree of polymerization
HDP: High degree of polymerization
ITS: Internal transcribed spacer
